# Further Advantages Conferred by the Amendment Act

**Published:** 1913-09-27

**Authors:** 


### OUR NATIONAL INSURANCE ACT DEPARTMENT.

### LXVIII.—Further Advantages Conferred by the Amendment Act.

Last week we dealt with some of the advantages conferred by the less known provisions of the Amendment Act, mentioning particularly those sections which, by simplifying the administrative machinery, are calculated to benefit the whole body of insured persons. This week we continue the subject, but direct attention chiefly to those provisions which relate to individuals and remove hardships that had become apparent from the practical application of the principal Act.

### A Maternity Benefit Anomaly Removed.

Under the Act of 1911 when a married woman is herself an insured person she is entitled on her confinement to sickness or disablement benefit as well as maternity benefit. If her husband is also insured the maternity benefit is payable from his insurance, and is claimed from his society, while the sickness or disablement benefit comes from her own insurance and is paid by her society. If the husband is not insured under the Act the maternity benefit is paid from her own insurance by her society as well as the sickness or disablement benefit.

One effect of these arrangements is that if for any reason the husband, although an insured person, should be out of benefit, no maternity benefit would be payable. The husband would be out of benefit, for instance, if he had not paid the requisite number of contributions, or if he were a deposit contributor, and had by reason of his own illness exhausted his credit in the deposit contributors' fund for the time being. In either case, in the particular circumstances, the fact of his being an insured person worked a hardship to the woman. If he had not been insured the maternity benefit would have been payable from her insurance, but because he was insured the claim could only be made against his insurance, with the result that, although contributions had been paid both by the husband and the wife, a smaller benefit would be received than if the wife only had been insured.

This is an anomaly that we pointed out several months ago, and we are glad to note that the Amendment Act contains provisions whereby that anomaly is removed. The section (Section 14 (2)) is designed to secure that the woman shall in no case be worse off because her husband is insured than she would be if he were not insured. If the husband is a member of an approved society, and either by reason of insufficiency or arrears of contribution is not entitled to maternity benefit from his insurance the benefit is to be paid by the woman's society from her insurance. If the husband is a deposit contributor and the amount standing to his credit is insufficient to pay the maternity benefit in full, the balance is to be made good to the woman from her own insurance.

This provision is to come into force on October 13, and in giving notice to approved societies to this effect the Insurance Commissioners point out that it will be encumbent upon the woman's society to make inquiries from the husband's society or from the deposit contributors' fund to ascertain if in fact the maternity benefit is not payable from such source. This would appear to involve delay in arriving at a settlement, but it is further emphasised by the Commissioners that there is no reason why such inquiries should delay the payment of benefits that would in any case come from the woman's society.

### Double Maternity Benefit.

In the foregoing statement we have spoken of the benefit payable to a woman from her own insurance, when her husband is insured, as sickness or disablement benefit. This is in accordance with the provisions of the Act of 1911, but the Amendment Act provides that in lieu of any sickness or disablement benefit to which she may be entitled under the principal Act she is to be entitled to receive a maternity benefit from her own insurance. The difference is of some importance, for although four weeks' sickness benefit at 7s. 6d. a week is the same in actual amount as a maternity benefit of 30s. the mode of payment is different, and in some cases it had been found that approved societies had declined payment of the sickness allowance unless supplied with a doctor's certificate of incapacity, which in some cases was difficult to obtain when the woman was attended by a midwife. By the new arrangement, which does not, however, come into operation until January 12 next, when a married woman is herself insured, the benefit payable on her confinement will be £3, half of which will in any case come from her society, and the balance either from her husband's insurance or her own, according as her husband is, or is not, an insured person. There is one qualification governing this concession, and that is that where such a sum is payable the woman must abstain from remunerative work during a period of four weeks after her confinement. It is partly for this reason that the section does not come into operation before January next, for rules have to be passed by the approved societies to the satisfaction of the Insurance Commissioners that such abstinence from remunerative work will be an essential requirement before payment is made. Although special facilities have been given by the Amendment Act for the authorisation of new rules consequent upon the passing of the Act, it was hardly feasible for the matter to be completed by all societies earlier than the date mentioned. While, therefore, we are disposed to regret that it was not possible to make the double maternity benefit regulations commence at an earlier date, the reason of the delay—namely, the adequate safeguard that women shall not go out to work for four weeks after confinement is so salutary that it is worth while waiting. It may, perhaps, be mentioned that it is the intention of the Insurance Commissioners to draw up a model rule, which may be adopted by the approved societies, and this should smooth the way for compliance with the regulations by the smaller societies.

New Privileges for Certain Aliens.

Aliens who are employed in an occupation which is employment within the meaning of the National Insurance Act have to be insured, but as a class they do not share the same benefits as British subjects. There was, however, an exception, for it was provided that an alien who on the date that the Bill was introduced (May 4, 1911) was a member of a society which, or a separate section of which, became an approved society and had then been resident in the United Kingdom for five years should be given the same privileges in regard to benefits as a British subject. This advantage is now extended by the Amendment Act to cover the case of an alien who was a member of a society which although not itself becoming an approved society "amalgamates with or transfers its engagements to an approved society, or which proves to the satisfaction of the Insurance Commissioners that it has organised, either solely or with other bodies, an approved society for the benefit of its members." It would seem that this provision was inserted in the Act to give legal sanction to the arrangement which we understand had been made with the large approved societies formed by the Collecting Friendly Societies and Industrial Assurance Offices, whereby their members who were able to satisfy the requirement of five years' residence were allowed by the Commissioners to be admitted to National Insurance benefits at British rates. Thus another anomaly has been removed by the Amendment Act.

There is still a further way in which aliens are benefited by the new Act. A woman who before marriage was a British subject by her marriage assumes her husband's nationality, and under the principal Act suffered the disqualification of her changed state. It was only upon her husband's death, or upon the dissolution or annulment of the marriage, or when she had for a period of two years been actually separated from or deserted by her husband that she could claim to be treated as a British subject. The disqualifications of the change of nationality, so far as they affect her insurance, are no longer to operate in any case. This provision of the Amendment Act, together with that last mentioned, are to come into operation on October 13, and all past contributions are to be reckoned as payments towards the benefits on the scale due to British subjects.

**NOTE.—Questions and Correspondence on all doubtful points are invited, and should be addressed to the Insurance Editor, The Hospital, 28 Southampton Street, Strand.**

